# Prevalence and Prognostic Importance of Massive Tricuspid Regurgitation in Patients Undergoing Tricuspid Annuloplasty With Concomitant Left-Sided Valve Surgery: A Study on Rheumatic Valvular Heart Disease

**DOI:** 10.3389/fcvm.2022.686208

**Published:** 2022-01-27

**Authors:** Yan Chen, Yap-Hang Chan, Mei-Zhen Wu, Yu-Juan Yu, Yui-Ming Lam, Ko-Yung Sit, Daniel Tai-Leung Chan, Cally Ka-Lai Ho, Lai-Ming Ho, Chu-Pak Lau, Wing-Kuk Au, Hung-Fat Tse, Kai-Hang Yiu

**Affiliations:** ^1^Department of Ultrasound, Shenzhen Hospital, Southern Medical University, Shenzhen, China; ^2^Division of Cardiology, Department of Medicine, The University of Hong Kong Shen Zhen Hospital, Shenzhen, China; ^3^Division of Cardiology, Department of Medicine, The University of Hong Kong, Queen Mary Hospital, Hong Kong, Hong Kong SAR, China; ^4^Department of Cardiothoracic Surgery, The University of Hong Kong, Queen Mary Hospital, Hong Kong, Hong Kong SAR, China; ^5^School of Public Health, The University of Hong Kong, Hong Kong, Hong Kong SAR, China

**Keywords:** tricuspid regurgitation (TR), tricuspid annuloplasty, effective regurgitant orifice area (EROA), adverse outcome, left-sided valve disease, rheumatic valvular heart disease

## Abstract

**Background:**

The presence of tricuspid regurgitation (TR) is very common in patients with concomitant left-sided valve disease. Recent studies have advocated an additional grading of massive TR that is beyond severe. The present study sought to characterize the spectrum of TR in patients undergoing tricuspid annuloplasty (TA) and to evaluate the prognostic value of TR severity for post-operative outcome following TA.

**Methods:**

A total of 176 patients who underwent TA with combined left-sided valve surgery, secondary to rheumatic valvular heart disease, were prospectively evaluated. The severity of TR was quantified by effective regurgitant orifice area (EROA) using the proximal isovelocity surface area method. Patients were categorized as having non-massive TR (EROA < 0.6 cm^2^) or massive TR (EROA ≥ 0.6 cm^2^). Adverse outcome was defined as all-cause mortality or heart failure requiring hospital admission following TA.

**Results:**

A total of 55 (31%) patients were considered to have massive TR. Patients with massive TR had a greater right ventricular dimension but a smaller left ventricular dimension compared with those with non-massive TR. After a median follow-up of 39 months, 35 adverse events occurred. Cox-regression analysis showed that both continuous EROA and dichotomized EROA (massive vs. non-massive TR) were independently associated with adverse events even after multivariable adjustment. Further, Harrell C index demonstrated that the addition of massive TR provided better discrimination ability of a prediction model to known prognosticators following TA.

**Conclusions:**

Massive TR is common and up to 31% of study population had massive TR. Massive TR was associated with adverse outcome in patients undergoing TA. Classification of the severity of TR by quantitative measures and identification of massive TR in patients with concomitant left-sided valve disease are essential when considering the optimal timing of corrective surgery.

## Introduction

Tricuspid regurgitation (TR) is a very common condition (1) that is closely associated with decreased survival (2). Traditionally, the severity of TR is classified as mild, moderate or severe assessed by transthoracic echocardiography. The measurement of effective regurgitant orifice area (EROA) has been advocated as a quantifiable assessment of TR grade and can provide superior prognostic value compared with qualitative and semi-quantitative assessment (3). Recent experience from patients referred for transcather tricuspid valve procedure has revealed that long-standing TR and regurgitant volume can double the conventional criteria for severe TR measured by EROA (4). Consequently, recent recommendation has further classified an extreme type of TR beyond severe, expanding the TR grading scheme to include “massive TR” and “torrential TR” (5).

Current guidelines (6, 7) recommend that tricuspid annuloplasty (TA) to correct TR is concomitant with left-side valve surgery, based on the clinical status of the left-sided valve lesion. The severity of TR in these patients is nonetheless not a consideration when determining timing of surgery. Indeed, TR is considered to run an indolent natural course and is not uncommon in patients with left-sided heart disease. Compared with severe TR, extreme severity of TR has recently been proven to be a strong predictor of adverse outcome, further supporting the need for another classification of extreme risk (8, 9). Nonetheless the prevalence of extreme TR, beyond severe, in patients undergoing TA during concomitant left-sided valve surgery is uncertain. In addition, the prognostic implication of extreme severity of TR in patients who underwent TA has not been evaluated. The present study aimed to characterize the spectrum of TR severity measured by EROA, in particular massive TR, in patients who underwent TA during concomitant left-sided valve surgery. The prognostic implication of TR severity for post-operative course following TA was also evaluated.

## Materials and Methods

### Study Population

This was a single-center prospective cohort study. The study was part of the Chinese Valvular Heart Disease Study (CVATS) to evaluate the pattern of disease, pathophysiology, and clinical outcome in Chinese patients (10). A total of 308 consecutive patients undergoing elective TA during left-sided valve surgery at Queen Mary Hospital between January 2013 and January 2019 were recruited. Patients were excluded if they had history of congenital heart disease (*n* = 7), pacemaker implantation (*n* = 9), or previous tricuspid valve surgery (*n* = 20). Patients were excluded if major lesion of the left-sided heart valve was recorded as non-rheumatic valvular heart disease (*n* = 84). Patients with poor-quality echocardiographic images (*n* = 12) that were unsuitable for further measure were also excluded. Accordingly, only 176 patients who underwent TA with combined left-sided valve surgery, secondary to rheumatic valvular heart disease, were included in the final analysis. Patients were followed up by one clinical investigator and details of adverse events were obtained from the electronic Clinical Management System. Adverse outcome was defined as all-cause mortality or heart failure requiring hospital admission following TA. Hospitalization for heart failure was defined as admission due to dyspnea with chest radiographic evidence of pulmonary congestion and treatment with intravenous diuretics. If patients had multiple adverse events, the first one was coded and recorded as study end point. For the study end points, patients who experienced adverse outcome were followed until the first episode of adverse event, the other patients were followed until February 2020. The study was approved by the institutional review board of Hospital Authority Hong Kong West Cluster and all participants gave written informed consent.

### Clinical Parameters

Baseline clinical information and laboratory blood tests for preoperative parameters were gathered at the time of recruitment. Conventional cardiovascular risk factors including diabetes mellitus, hypertension, hyperlipidemia and smoking status were recorded. New York Heart Association (NYHA) classification was recorded as class I/II or class III/IV, and the status of valvular atrial fibrillation (AF) was also retrieved for each subject from Hospital Authority records. Detailed surgery type including coronary artery bypass grafting and combined left-sided valve surgery during TA surgery was recorded. Data on prescription of angiotensin converting enzyme inhibitor (ACEI) or angiotensin receptor blocker (ARB), calcium-channel, blocker beta-blocker and statin were also collected. The European System for Cardiac Operative Risk Evaluation (EuroSCORE II) was employed to estimate operative mortality risk.

### Echocardiography Parameters

Comprehensive transthoracic echocardiography was performed before valvular surgery using GE Vivid E9 echocardiography system. All image acquisitions were recorded over three consecutive cycles. Left ventricular (LV) and right ventricular (RV) echocardiographic parameters including left ventricular end-diastolic volume (LVEDV), left ventricular end-systolic volume (LVESV), left ventricular ejection fraction (LVEF), right ventricular end-diastolic area (RVEDA), right ventricular end-systolic area (RVESA), and right ventricular fractional area change (RVFAC) were measured according to the current recommendations (11). Tricuspid annulus diameter was measured from the insertion of the septal leaflet to the insertion of the anterior leaflet at end-diastole. Tricuspid annular plane systolic excursion (TAPSE), a measure of RV systolic function, was obtained from the M-mode apical four-chamber view. Pulmonary arterial systolic pressure (PASP) was estimated from peak TR velocity by continuous-wave Doppler using the modified Bernoulli equation: PASP = 4(V)^2^ + right atrial pressure value (12).

The severity of TR was quantified by effective regurgitant orifice area (EROA) using the proximal isovelocity surface area (PISA) method (13, 14). The PISA method was used to calculate EROA by combining the measurement of TR flow and its velocity by continuous-wave Doppler, as previously described (13, 14). As shown in our previous study (15): color Doppler images of TR proximal flow convergence were obtained from apical 4-chamber views and zoomed to the region of interest, the color-flow velocity scale was maximized and the baseline was shifted downwards until the flow convergence region was visualized clearly. The Nyquist limit (aliasing velocity) was controlled at 0.28–0.34 m/s in order to optimize visualization and avoid overestimation or underestimation under color Doppler. Radial distance between the first aliasing velocity (blue/yellow interface) and the center of the tricuspid orifice was measured in mid-systole to calculate regurgitant flow, and the EROA was then calculated as the ratio of regurgitant flow to the peak velocity of the TR jet (15). Patients were divided into two groups based on their TR severity according to the recommendation (5): 121 patients with non-massive TR (EROA <0.6 cm^2^) and 55 patients with massive TR (EROA ≥ 0.6 cm^2^). Residual significant TR was defined as moderate or severe TR according to transthoracic echocardiography examination results before discharge after TA surgery.

### Statistical Analysis

Continuous variables are expressed as mean ± SD if normally distributed or median (25–75th percentiles) if non-normally distributed. Categorical variables are described as numbers (percentages). Student *t*-test and Mann-Whitney *U*-test were used to compare continuous variables between two groups. Categorical variables were compared using Chi-square test or Fisher's exact test. Univariate Cox regression analysis was performed to evaluate the potential predictors of long-term adverse outcome. Multivariable Cox regression analysis was subsequently performed to determine the independent predictive ability of EROA for long-term adverse outcome. The Harrell C statistic was calculated using Stata 14.0 to assess the prediction value of each primary model and comparison model for long-term adverse outcome. The higher Harrell C index indicated that the better the model can discriminate the adverse outcome. The incremental prognostic value of massive TR was subsequently assessed in nested Cox regression model that includes the other risk factors. To compare the adverse outcome for massive and non-massive TR, Kaplan-Meier curve was constructed and the percentage of adverse events compared using the log-rank test. All statistical analyses were performed using the statistical package SPSS (Version 22.0, SPSS, Chicago, USA) and *P*-values reported are 2-sided for consistency. A *P*-value < 0.05 was considered statistically significant.

## Results

### Baseline Characteristics

The baseline characteristics of the entire study cohort and patients with and without massive TR are shown in Table 1. All the patients had functional TR secondary to left-sided rheumatic valvular heart disease, and underwent TA with ring annuloplasty. For the entire study population, up to 85% of study population had AF. The median EuroSCORE II was 3.2% (interquartile range: 1.9–5.4%). Pre-operative mean LVEF and RVFAC were respectively 60 and 48%, suggesting a preserved LV and RV function in the study population. Further, the median EROA was 0.40 cm^2^ (interquartile range: 0.25–0.66 cm^2^) and 55 (31%) patients were considered to have massive TR. The mean tricuspid annulus diameter and PASP was 3.7 ± 0.6 cm and 47.9 ± 12.6 mmHg, respectively. The most common combined left-sided valve procedure during TA was mitral valve replacement. All patients experienced cardiopulmonary bypass and 10 patients who had significant coronary artery disease, underwent simultaneous coronary artery bypass grafting.

**Table 1:** Baseline characteristics of the study population.

**Variables**	**Overall**	**Non-massive TR**	**Massive TR**	***P*-value**
	**(*n* = 176)**	**(EROA <0.6 cm^**2**^, *n* = 121)**	**(EROA ≥0.6 cm^**2**^, *n* = 55)**	
Age (years)	64.4 ± 8.2	63.8 ± 8.5	65.9 ± 7.3	0.10
Male, *n* (%)	44 (25.0)	25 (20.7)	19 (34.5)	0.05
Diabetes mellitus, n (%)	33 (18.8)	20 (16.5)	13 (23.6)	0.26
Hypertension, *n* (%)	28 (15.9)	18 (14.9)	10 (18.0)	0.58
Hyperlipidemia, *n* (%)	33 (18.8)	22 (18.2)	11 (20.0)	0.78
Smoking, *n* (%)	24 (13.6)	16 (13.2)	8 (14.5)	0.81
Atrial fibrillation, *n* (%)	150 (85.2)	102 (84.3)	48 (87.3)	0.61
NYHA class III/IV, *n* (%)	75 (42.6)	49 (40.5)	26 (47.3)	0.40
Hemoglobin (g/dL)	12.3 ± 1.8	12.6 ± 1.7	11.8 ± 1.9	0.01
eGFR (mL/min/1.73 m^2^)	71.4 ± 18.7	73.6 ± 17.4	66.4 ± 20.5	0.02
**Combined valvular surgery with TA**, ***n*** **(%)**				
Mitral valve repair	15 (8.5)	11 (9.1)	4 (7.3)	0.78
Mitral valve replacement	78 (44.3)	53 (43.8)	25 (45.5)	0.84
Aortic valve replacement	17 (9.7)	9 (7.4)	8 (14.5)	0.14
Dual valvular surgery	66 (37.5)	48 (39.7)	18 (32.7)	0.38
Concomitant CABG, *n* (%)	10 (5.7)	5 (4.1)	5 (9.1)	0.29
**Medications**, ***n*** **(%)**				
ACEI/ARB	64 (36.4)	42 (34.7)	22 (40.0)	0.50
Beta blocker	67 (38.1)	44 (36.4)	23 (41.8)	0.49
Calcium-channel blockers	41 (23.3)	29 (24.0)	12 (21.8)	0.76
Statins	51 (29.0)	36 (29.8)	15 (27.3)	0.74
EuroSCORE II (%)	3.2 (1.9–5.4)	3.0 (1.8–5.1)	4.0 (2.4–7.0)	0.02
**Echocardiographic parameters**				
LVEDV (ml)	80.0 (62.0–101.8)	83.0 (68.5–106.0)	72.0 (55.0–94.0)	0.03
LVESV (ml)	31.0 (23.0–43.0)	32.0 (25.0–44.0)	27.0 (20.0–41.0)	0.09
LVEF (%)	59.6 ± 8.0	59.7 ± 8.1	59.5 ± 7.8	0.93
RVEDA (cm^2^)	14.5 (11.9–19.0)	12.9 (11.1–15.8)	19.4 (15.8–23.4)	<0.01
RVESA (cm^2^)	7.4 (5.7–9.7)	6.6 (5.4–8.3)	10.3 (7.9–12.9)	<0.01
RVFAC (%)	48.2 ± 7.6	49.1 ± 6.8	46.2 ± 8.8	0.04
TAPSE (cm)	1.6 ± 0.3	1.7 ± 0.3	1.5 ± 0.2	<0.01
Tricuspid annulus diameter (cm)	3.7 ± 0.6	3.5 ± 0.5	4.2 ± 0.6	<0.01
PASP (mmHg)	47.9 ± 12.6	48.4 ± 12.2	46.7 ± 13.4	0.41
EROA (cm^2^)	0.40 (0.25–0.66)	0.29 (0.21–0.40)	0.76 (0.68–1.20)	<0.01
Residual significant TR, *n* (%)	14 (8.0)	9 (7.4)	5 (9.1)	0.77

*Values are mean ± SD or median (25–75th percentiles), or n (%)*.

*ACEI, angiotensin converting enzyme inhibitor; ARB, angiotensin receptor blocker; CABG, coronary artery bypass graft; eGFR, estimated glomerular filtration rate; LVEDV, Left ventricular end-diastolic volume; LVEF, left ventricular ejection fraction; LVESV, Left ventricular end-systolic volume; NYHA, New York Heart Association; PASP, pulmonary artery systolic pressure; RVEDA, right ventricular end-diastolic area; RVESA, right ventricular end-systolic area; RVFAC, right ventricular fractional area change; TA, tricuspid annuloplasty; TAPSE, tricuspid annular plane systolic excursion; TR, tricuspid regurgitation; EROA, effective regurgitant orifice area; EuroSCORE, European System for Cardiac Operative Risk Evaluation*.

Patients with massive TR had lower hemoglobin and estimated glomerular filtration rate, higher EuroSCORE II (all *P* < 0.05). As expected, patients with massive TR had larger RVEDA [19.4 (15.8–23.4) vs. 12.9 (11.1–15.8) cm^2^, *P* < 0.01], RVESA [10.3 (7.9–12.9) vs. 6.6 (5.4–8.3) cm^2^, *P* < 0.01], and tricuspid annulus diameter (4.2 ± 0.6 vs. 3.5 ± 0.5 cm, *P* < 0.01), and lower RVFAC (46.2 ± 8.8 vs. 49.1 ± 6.8%, *P* = 0.04) and TAPSE (1.5 ± 0.2 vs. 1.7 ± 0.3 cm, *P* < 0.01) compared with those with non-massive TR. In contrast, LVEDV [72.0 (55.0–94.0) vs. 83.0 (68.5–106.0) ml, *P* = 0.03] was significantly smaller in patients with massive TR compared with those with non-massive TR. The other clinical parameters were nonetheless similar between the two groups (Table 1).

### Predictors Associated With Long-Term Adverse Outcome

Median follow-up following TA was 39 months (range 1–86 months). A total of 35 adverse events happened: including 19 hospitalizations for heart failure (nine in non-massive TR and 10 in massive TR, 7.7 vs. 23.3%, Log-rank test *P* = 0.015) and 16 deaths (four in non-massive and 12 in massive TR, 3.6 vs. 26.7%, Log-rank test *P* < 0.001). Univariate Cox regression analysis of baseline characteristics associated with long-term adverse events are shown in Table 2. Clinical parameters including older age, male gender, diabetes mellitus, hypertension, advanced NYHA class, lower hemoglobin and estimated glomerular filtration rate, higher EuroSCORE II were associated with adverse events. Regarding echocardiographic parameters, a larger RVEDA, RVESA, tricuspid annulus diameter, a higher RVFAC and lower TAPSE were associated with adverse outcome (Table 2). Importantly, both EROA (as a continuous variable) and categorical variable of EROA (massive TR vs. non-massive TR) were correlated with adverse outcome. Nonetheless LV volume and ejection fraction showed no such relationship.

**Table 2:** Factors associated with long-term adverse events by univariate Cox regression analysis.

**Variables**	**Univariate analysis**		
	**HR**	**95% CI**	***P***
Age	1.06	1.02–1.11	<0.01
Male	2.84	1.46–5.54	<0.01
Diabetes mellitus	2.08	1.02–4.25	0.04
Hypertension	3.20	1.59–6.44	<0.01
Hyperlipidemia	1.38	0.63–3.03	0.43
Smoking	1.84	0.80–4.22	0.15
Atrial fibrillation	1.43	0.50–4.05	0.50
NYHA class III/IV	2.08	1.06–4.07	0.03
Hemoglobin	0.73	0.61–0.86	<0.01
eGFR	0.97	0.95–0.99	<0.01
**Combined valvular surgery with TA**			
Mitral valve repair	1.93	0.75–4.97	0.17
Mitral valve replacement	0.84	0.43–1.65	0.61
Aortic valve replacement	1.15	0.41–3.25	0.79
Dual valvular surgery	0.87	0.43–1.74	0.68
Concomitant CABG	0.91	0.22–3.79	0.89
**Medications**			
ACEI/ARB	1.92	0.99–3.73	0.06
Beta blocker	1.06	0.54–2.08	0.87
Calcium-channel blockers	0.68	0.30–1.56	0.36
Statins	0.80	0.38–1.71	0.57
EuroSCORE II	1.10	1.06–1.14	<0.01
LVEDV	1.01	0.99–1.01	0.23
LVESV	1.01	0.99–1.02	0.40
LVEF	1.01	0.96–1.05	0.83
RVEDA	1.08	1.03–1.14	<0.01
RVESA	1.13	1.05–1.21	<0.01
RVFAC	0.96	0.92–0.99	0.02
TAPSE	0.28	0.08–0.98	<0.05
Tricuspid annulus diameter	1.93	1.22–3.06	<0.01
PASP	1.01	0.99–1.04	0.25
EROA (per 0.1 cm^2^ increase)	1.59	1.29–1.96	<0.01
Massive TR vs. non-massive TR	4.05	2.04–8.03	<0.01
Residual significant TR	1.64	0.58–4.65	0.35

*ACEI, angiotensin converting enzyme inhibitor; ARB, angiotensin receptor blocker; CABG, coronary artery bypass graft; eGFR, estimated glomerular filtration rate; LVEDV, Left ventricular end-diastolic volume; LVEF, left ventricular ejection fraction; LVESV, Left ventricular end-systolic volume; NYHA, New York Heart Association; PASP, pulmonary artery systolic pressure; RVEDA, right ventricular end-diastolic area; RVESA, right ventricular end-systolic area; RVFAC, right ventricular fractional area change; TA, tricuspid annuloplasty; TAPSE, tricuspid annular plane systolic excursion; TR, tricuspid regurgitation; EROA, effective regurgitant orifice area; EuroSCORE, European System for Cardiac Operative Risk Evaluation*.

### Independent Predictive Ability of EROA for Long-Term Adverse Events

**Figure 1:**
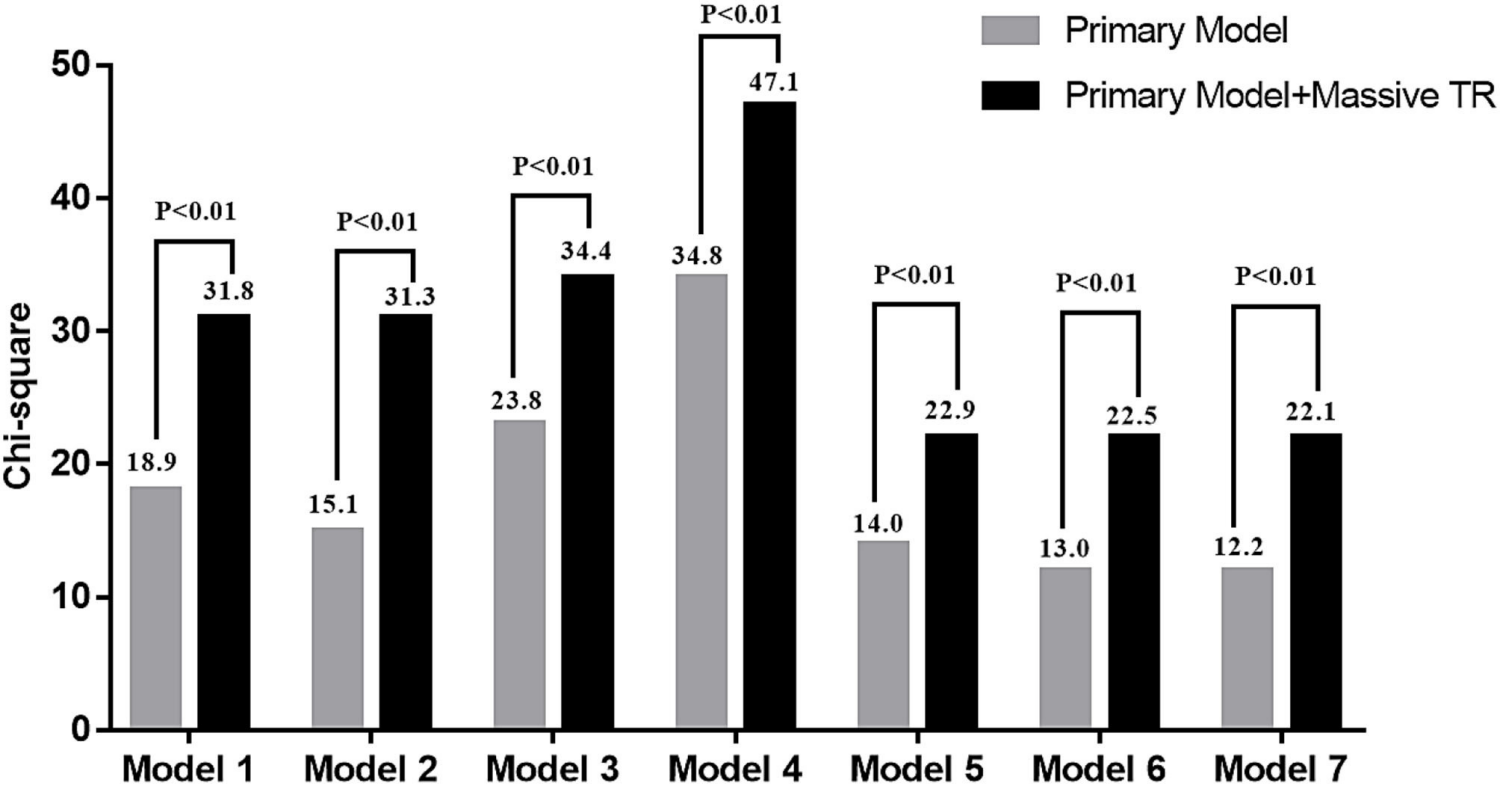
Change in model Chi square with addition of massive tricuspid regurgitation (TR) to the other risk factors. The including parameters of each primary model was same as Table 3.

As shown in Table 3, multivariable Cox regression analysis showed that EROA (as a continuous variable) was independently associated with adverse events, even after adjusting for the other potential risk factors. In addition, patients with massive TR had a 3-fold risk of developing adverse events compared with patients with non-massive TR. Importantly, Harrell C index demonstrated that adding dichotomized EROA assessment provided better discrimination of a prediction model in each comparison model (Table 4). Furthermore, nested Cox regression analysis showed that the addition of massive TR provided incremental prognostic value beyond demographic parameters, traditional cardiovascular risk factors, clinical data and important echocardiographic parameters (Figure 1).

**Table 3:** Prognostic value of TR severity.

	**TR-EROA (per 0.1 cm**^**2**^ **increase)**		**Massive TR vs. non-massive TR**	
	**HR (95% CI)**	***P*-value**	**HR (95% CI)**	***P*-value**
Model 1	1.35 (1.08–1.69)	<0.01	3.16 (1.57–6.37)	<0.01
Model 2	1.69 (1.36–2.11)	<0.01	3.67 (1.84–7.32)	<0.01
Model 3	1.36 (1.09–1.70)	<0.01	2.94 (1.44–6.02)	<0.01
Model 4	1.62 (1.29–2.03)	<0.01	3.89 (1.94–7.82)	<0.01
Model 5	1.52 (1.06–2.18)	0.02	3.09 (1.40–6.79)	<0.01
Model 6	1.59 (1.11–2.26)	0.01	3.26 (1.49–7.12)	<0.01
Model 7	1.49 (1.10–2.01)	0.01	3.30 (1.44–7.55)	<0.01

*Model 1 = adjusted for demographic parameters including age, male*.

*Model 2 = adjusted for traditional cardiovascular risk factors including diabetes mellitus, hypertension*.

*Model 3 = adjusted for blood biochemical parameters including hemoglobin, eGFR*.

*Model 4 = adjusted for NYHA class III/IV, EuroSCORE II*.

*Model 5 = adjusted for echocardiographic parameters including RVEDA, RVFAC, TAPSE*.

*Model 6 = adjusted for echocardiographic parameters including RVESA, RVFAC, TAPSE*.

*Model 7 = adjusted for echocardiographic parameters including tricuspid annulus diameter, RVFAC and TAPSE*.

*RVEDA, RVESA and tricuspid annulus diameter were collinearity. To avoid bias from multicollinearity and follow the statistical rules, RVEDA, RVESA, and tricuspid annulus diameter were entered into multivariable analysis individually*.

*CI, confidence interval; eGFR, estimated glomerular filtration rate; HR, hazard ratio; NYHA, New York Heart Association; RVEDA, right ventricular end-diastolic area; RVESA, right ventricular end-systolic area; RVFAC, right ventricular fractional area change; TAPSE, tricuspid annular plane systolic excursion; TR, tricuspid regurgitation; EROA, effective regurgitant orifice area; EuroSCORE, European System for Cardiac Operative Risk Evaluation*.

**Table 4:** Better discrimination of a prediction model after including dichotomized EROA.

	**Harrell C index (95% CI)**	
	**Primary**	**Comparison**
	**model**	**model**
Model 1 vs. Model 1+ dichotomized EROA*	0.68 (0.58–0.79)	0.74 (0.67–0.82)
Model 2 vs. Model 2+ dichotomized EROA*	0.72 (0.60–0.83)	0.79 (0.71–0.86)
Model 3 vs. Model 3+ dichotomized EROA*	0.73 (0.65–0.82)	0.76 (0.69–0.84)
Model 4 vs. Model 4+ dichotomized EROA*	0.71 (0.63–0.80)	0.78 (0.72–0.85)
Model 5 vs. Model 5+ dichotomized EROA*	0.68 (0.60–0.77)	0.71 (0.62–0.79)
Model 6 vs. Model 6+ dichotomized EROA*	0.67 (0.59–0.76)	0.70 (0.61–0.80)
Model 7 vs. Model 7+ dichotomized EROA*	0.67 (0.59–0.75)	0.70 (0.61–0.79)

*Model 1 = age, male*.

*Model 2 = diabetes mellitus, hypertension*.

*Model 3 = hemoglobin, eGFR*.

*Model 4 = NYHA class III/IV, EuroSCORE II*.

*Model 5 = RVEDA, RVFAC, TAPSE*.

*Model 6 = RVESA, RVFAC, TAPSE*.

*Model 7 = Tricuspid annulus diameter, RVFAC and TAPSE*.

Dichotomized EROA*: Massive TR vs. non-massive TR (EROA ≥ 0.6 cm^2^ vs. EROA <0.6 cm^2^).

*CI, confidence interval; eGFR, estimated glomerular filtration rate; NYHA, New York Heart Association; RVEDA, right ventricular end-diastolic area; RVESA, right ventricular end-systolic area; RVFAC, right ventricular fractional area change; TAPSE, tricuspid annular plane systolic excursion; TR, tricuspid regurgitation; EROA, effective regurgitant orifice area; EuroSCORE, European System for Cardiac Operative Risk Evaluation*.

### Comparison of Adverse Events for Massive TR and Non-massive TR

**Figure 2:**
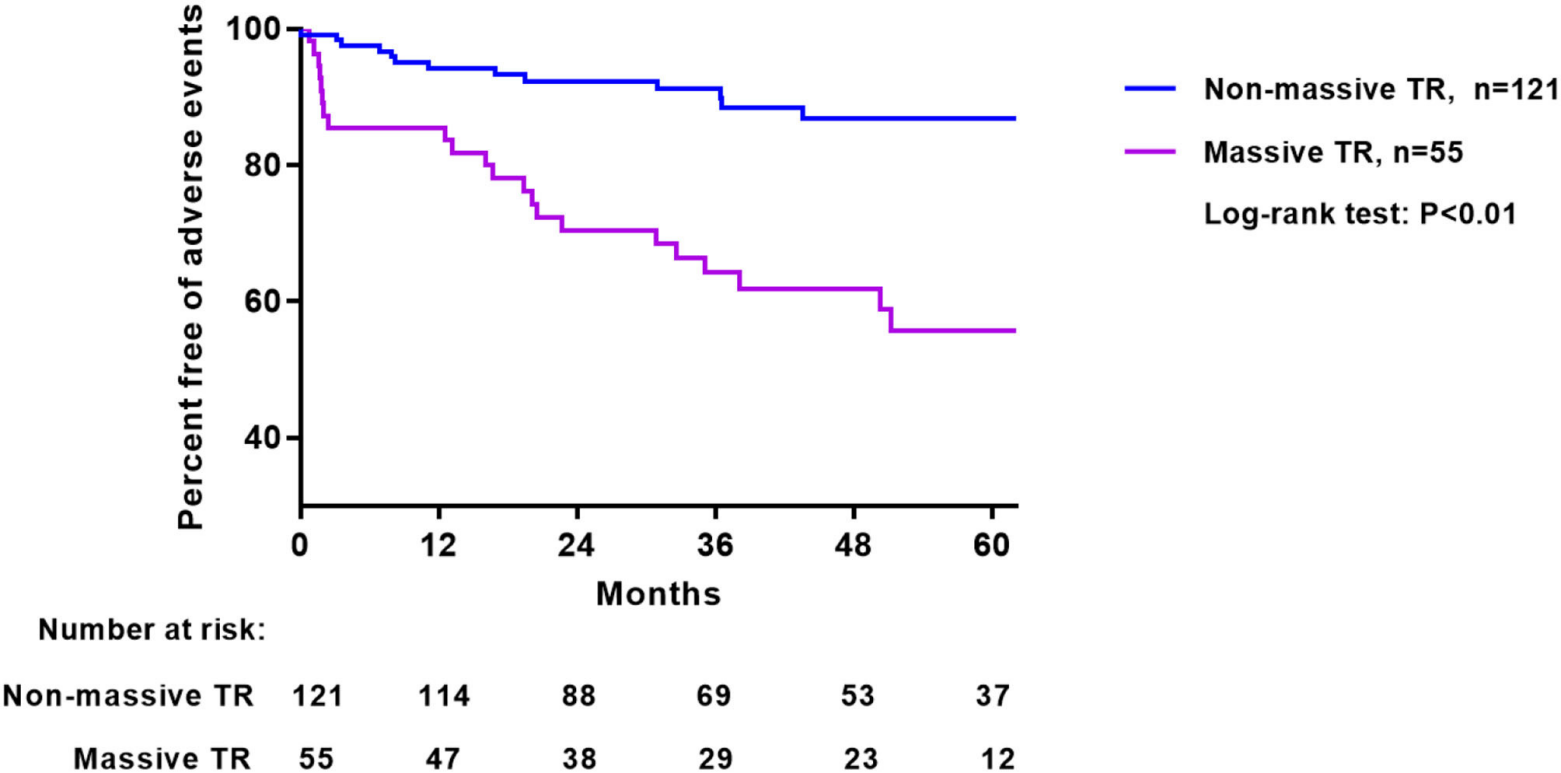
Kaplan-Meier analysis according to tricuspid regurgitation (TR) severity.

The Kaplan-Meier survival curve analysis showed that patients with massive TR had a significantly higher percentage of adverse outcome than those with non-massive TR (Figure 2). The incidence of adverse outcome was 15% at 1 year, 30% at 2 years, and 36% at 3 years for patients with massive TR, significantly higher compared with that for patients with non-massive TR (6% at 1 year, 8% at 2 years, and 10% at 3 years).

## Discussion

Our study demonstrates that in patients undergoing TA during concomitant left-sided valve surgery, the prevalence of massive TR, defined as an EROA ≥ 0.6 cm^2^, is not uncommon and present in nearly a third of patients. Of interest, despite having a greater RV dimension and more impaired RV function, patients with massive TR had a smaller LV dimension, and a similar LV ejection fraction and NYHA functional class compared with those with non-massive TR. Importantly, patients with massive TR had a worse outcome following TA than those with non-massive TR. The Harrell C index analysis further revealed that the addition of massive TR provided better discrimination power of perdition model for adverse events compared with other known prognostic factors.

### Prevalence of Massive TR

The presence of TR is often an incidental finding during echocardiographic assessment. A trivial or mild degree is generally regarded as benign. In contrast, those with moderate and severe TR have an increased risk of adverse outcome (2). In the Framingham Heart Study, the prevalence of moderate and severe TR was up to 1.5 and 5.6% in men and women aged >70 years, respectively (1). In another study that evaluated over 5,000 adults at three Veterans Affairs medical centers, moderate or severe TR was present in 15.7% (2). In the OxVALVE population cohort study that included 2,500 individuals aged ≥65 years with no known valvular disease, moderate/severe TR was present in 2.9% and was the most common valvular lesion detected (16). Collectively, these studies suggest that significant TR is not uncommon in clinical practice and undoubtedly deserves more attention (17). Recent studies have demonstrated that late in the natural history of the disease, patients may further develop an extreme form of TR, beyond the current definition of severe grade (18). As a result, there has been a move to revise the current TR grading, expanding the spectrum beyond severe to include massive and torrential (5). Further, symptoms in patients with TR, such as ankle oedema, are usually well-tolerated and generally respond to diuretic therapy. It is only at a later stage that TR may cause symptoms of right-sided heart failure such as weight loss, ascites and cachexia. Because of these insidious and non-specific symptoms, the presence of significant TR can sometimes be overlooked. In our present study, we determined that massive TR can occur in nearly a third of patients with concomitant left-sided valve disease. Indeed, massive TR is even more frequent in patients undergoing percutaneous tricuspid valve intervention who are considered too high risk for conventional surgery (4). These findings highlight the increasing prevalence of massive TR that will place a significant health burden on society parallel to our aging population. Large epidemiological studies are required to further evaluate the prevalence of extreme forms of TR that perhaps are asymptomatic and remain unidentified or underestimated.

### Characteristics of Patients With Massive TR

In our present study, patients with massive TR exhibited a higher EuroSCORE II compared with those with non-massive TR. This illustrates that patients with massive TR are more likely to have advanced diseases with clustering of comorbidities. Nonetheless the NYHA class was similar between the two groups of patients, indicating that subjective functional assessment cannot accurately describe the complex risk profile. This finding further underscores that the timing of surgery, driven partly by symptoms, cannot distinguish the composite risk of patients with and without massive TR.

In addition, our finding reveals that although RV dimension was larger, patients with massive TR had a smaller LV dimension compared with those with non-massive TR. This intriguing observation is consistent with another study that revealed LV dimension to be inversely correlated with severity of TR in a cohort of patients with LV systolic dysfunction (19). One possible explanation could be that pre-load of the LV is smaller in patients with massive TR, leading to smaller LV. Another reason could speculate that the smaller LV dimension observed in those with more severe TR was due to compression by the enlarged RV within a confined pericardial space. As a result, the paradoxically smaller LV dimension may create a false impression to the clinician that patients with massive TR have a preserved LV dimension, despite having a higher EuroSCORE II. Given that LV dilatation is a key factor that determines the timing of surgery, the misinterpretation of a preserved LV dimension in patients with massive TR may further delay surgery. Studies to evaluate the optimal cut-off value of LV dimension or the LV eccentricity index (20), an index that reflects abnormal motion of the interventricular septum due to RV volume overload, to predict adverse outcome in patients with massive TR would nonetheless require clarification by future studies.

### Prognostic Implication of Massive TR

The TR has often been considered a forgotten entity of valve disease, in part due to its secondary nature in left-sided valve disease and long latent asymptomatic period. Increasingly, studies have now demonstrated that TR is not a benign entity and the presence of moderate and severe TR is correlated with adverse outcome (2, 19). A recent study further demonstrated that patients with an extreme degree of TR with an EROA > 0.7 cm^2^ exhibited poorer survival than those with severe TR (EROA >0.4 and <0.7 cm^2^) (8). Similarly, in a study that recruited consecutive severe TR patients, massive TR (EROA ≥ 0.6 cm^2^) was associated with mortality and heart failure re-hospitalization (9). These studies reiterate the need to revise TR grading. The current definition of severe TR cannot completely accentuate the dismal outcome for those with an extreme form of TR. Our present findings further demonstrate that patients with massive TR experience an adverse outcome following TA. It is clear that early surgical correction before the development of massive TR is warranted as well as intense clinical surveillance following surgery in order to improve clinical performance.

### Clinical Implications

The prevalence of TR is increasing, with an estimated prevalence of around 1.6 million patients with significant TR in the USA. Nonetheless fewer than 8,000 patients undergo tricuspid valve surgery per year (21). One reason for this relatively small number of corrective surgeries is perhaps the controversial optimal timing of surgical intervention for TR, mostly due to the limited data available and their heterogeneous nature (6). Another reason may be the poor outcomes following surgery due to late referrals that are often associated with a high risk condition such as hepatic or renal dysfunction. Delayed surgical correction for patients with significant TR may explain their poor long-term postoperative mortality with 5- and 10-year survival rates of 62–72 and 49–51%, respectively (22–24).

According to the current guidelines, TA should be performed during concomitant left-sided valve surgery in patients with TR or dilated tricuspid annulus (6, 7). Recent research further showed that the inclusion of TA at the time of mitral valve surgery resulted in a lower risk of a primary-end-point event at 2 years than those who underwent mitral-valve surgery alone (25). Nonetheless the severity of TR, in particular massive TR, is not one of the indications for surgical correction. Our study highlights that a significant proportion of patients who undergo TA during concomitant left-sided valve surgery have already developed massive TR that is strongly associated with adverse events. Surgeons should consider earlier TA before the development of massive TR, even when LV remodeling has not reached a level that warrants left-sided valve surgery, in order to optimize clinical outcome. Future studies are warranted to provide confirmatory evidence and to support the assessment of TR severity as a factor determining timing of surgery.

### Limitations

There are several limitations in this study. Because of the single center study and small number of patients with extreme TR, we used only EROA ≥ 0.6 cm^2^ to define massive TR. A larger study population is required to further characterize the prognostic risk for those with torrential TR (EROA ≥ 0.8 cm^2^) according to the recommendation (5). Further, a larger sample size is required to better discriminate the adverse outcome and define the cut-off value of EROA to predict adverse outcome. The present study only included patients who underwent TA with combined left-sided valve surgery; future study should be verified in patients undergoing isolated TA and in a control group. The advent of three-dimensional assessment-derived EROA or vena contracta area may improve the quantification of TR severity (26), and advanced speckle tracking analysis derived right ventricular strain may provide additional predictive information. Therefore, their prognostic value in patients undergoing TA will require future evaluation. Post-operative detailed echocardiography was not systematically performed and post-intervention EROA would require evaluation by future prospective study. In order to confirm the clinical benefits of early TA prior to the development of massive TR, a prospective, randomized multicenter trial is required. In addition, right heart catheter was not performed routinely in our locality and thus invasive measurement of pulmonary hypertension cannot be systematically evaluated. Furthermore, the present study mainly represents Chinese rheumatic valvular heart disease and thus these findings should be confirmed by European/US patients, who may experience functional TR due to non-rheumatic left-sided valve disease.

## Conclusion

This study provides novel evidence that massive TR is common in patients who underwent TA during concomitant left-sided valve surgery. Importantly, massive TR is independently associated with a dismal outcome. Our observations provide evidence to support the notion that as a coexisting entity, TR severity should be considered to determine the optimal timing for surgery in patients with concomitant left-sided valve disease.

## Data Availability Statement

The original contributions presented in the study are included in the article/supplementary material, further inquiries can be directed to the corresponding author/s.

## Ethics Statement

The studies involving human participants were reviewed and approved by the Ethics Committee of the West Cluster Hospital Authority of Hong Kong. The patients/participants provided their written informed consent to participate in this study.

## Author Contributions

YC and K-HY contributed to conception and design of the study, collection, analysis and interpretation of the data, and drafting and revising of the manuscript. Y-HC, M-ZW, Y-JY, Y-ML, K-YS, DC, CH, and L-MH contributed to patients' recruitment, data collection, and data analysis. C-PL, W-KA, and H-FT contributed to conception of the study and revising of the manuscript. All authors have significant contribution to the manuscript and have approved the final version for submission.

## Conflict of Interest

The authors declare that the research was conducted in the absence of any commercial or financial relationships that could be construed as a potential conflict of interest.

## Publisher's Note

All claims expressed in this article are solely those of the authors and do not necessarily represent those of their affiliated organizations, or those of the publisher, the editors and the reviewers. Any product that may be evaluated in this article, or claim that may be made by its manufacturer, is not guaranteed or endorsed by the publisher.
